# Global Transcriptional Analysis of Yeast Cell Death Induced by Mutation of Sister Chromatid Cohesin

**DOI:** 10.1155/2008/634283

**Published:** 2008-06-09

**Authors:** Qun Ren, Hui Yang, Bifeng Gao, Zhaojie Zhang

**Affiliations:** ^1^Department of Zoology and Physiology, University of Wyoming, Laramie, WY 82071, USA; ^2^Microarray Core, University of Colorado Health Science Center, Denver, CO 80262, USA

## Abstract

Cohesin is a protein complex that regulates sister chromatid cohesin during cell division. Malfunction in chromatid cohesin results in chromosome missegregation and aneuploidy. Here, we report that mutations of MCD1 and PDS5, two major components of cohesin in budding yeast, cause apoptotic cell death, which is characterized by externalization of phosphatidylserine at cytoplasmic membrane, chromatin condensation and fragmentation, and ROS production. Microarray analysis suggests that the cell death caused by mutation of MCD1 or PDS5 is due to the internal stress response, contrasting to the environmental or external stress response induced by external stimuli, such as hydrogen peroxide. A common feature shared by the internal stress response and external stress response is the response to stimulus, including response to DNA damage, mitochondria functions, and oxidative stress, which play an important role in yeast apoptotic cell death.

## 1. Introduction

It has been increasingly evident that yeast as well as other unicellular organisms can undergo apoptotic cell death (for recent review, see [1]).The apoptotic cell death shares many morphological and biochemical features with mammalian apoptosis, including DNA fragmentation and chromatin condensation, exposure of phosphatidylserine on the outer membrane surface, leakage of Cytochrome C from mitochondria [2]. Apoptosis in yeast can be induced by external stresses, such as hydrogen peroxide [3], acetic acid [4], as well as mutations of certain essential genes, such as CDC48 [5], ORC2 [6], and PDS5 [7]. The yeast apoptotic cell death is promoted by proapoptotic genes, including the metacaspase YCA1/MCA1 [8, 9] and apoptosis-inducing factor AIF1 [10].

Cohesin is a protein complex that regulates sister chromatid cohesion and is required for ordered chromosome segregation [11]. Cohesin physically links the sister chromatids during replication. This attachment makes it possible for the kinetochore of each sister chromatid to attach to microtubules from different poles. During anaphase, cohesin is released from chromosomes, and proper segregation occurs [12]. Cohesin contains at least four protein subunits: Smc1, Smc3, Mcd1/Scc1, and Scc3 [13, 14]. In addition to the four subunits, several additional proteins are required to establish or maintain the chromatid cohesion but are not part of the cohesin complex, such as Pds5 [14–17], Scc2, and Scc4 [18]. Scc2 and Scc4, for example, are required for loading the cohesin complex onto chromosomes, but do not colocalize with the complex [18]. Pds5 is essential for both mitosis [15, 16, 19] and meiosis [20]. Pds5 localizes to chromosomes in a cell cycle-dependent manner and is required for sister chromatid cohesion, chromosome condensation, DNA repair, and proper chromosome segregation.

In addition to its function in chromatid cohesion, studies show that cohesin has a role in apoptosis [21, 22]. Human RAD21/MCD1 is reported as a nuclear caspase target. Induction of apoptosis by diverse stimuli causes the cleavage of hRad21. The cleaved C-terminal product of hRAD21 is translocated from the nucleus to cytoplasm and acts as a nuclear signal for apoptosis. RAD21/MCD1 is also overexpressed in prostate [23] and breast cancer cells [24]. Suppression of RAD21/MCD1 reduces the ability for cell proliferation and increases the level of apoptosis [24]. In yeast (S. cerevisiae), our recent study showed that mutation of PDS5 causes cell death in early meiosis [7].

Here we report that mutation of MCD1 (mcd1-1) and PDS5 (pds5-1) showed an apoptotic phenotype, including exposure of phosphatidylserine to the outer membrane, accumulation of DNA breaks, condensation and fragmentation of chromatin, and production of ROS. Microarray analysis of the global transcript of mcd1-1 and pds5-1 mutants, in comparison with H_2_O_2_ induced apoptosis, showed that the majority of the differentially expressed genes were upregulated. These genes were involved in a variety of functions, including response to stimulus, cell cycle regulation, mitochondrial functions, and cell wall organization. Our data suggest that the cell death caused by mutation of MCD1 or PDS5 is due to response to physiological or internal stress, compared to the H_2_O_2_-treated cells, which is considered response to environmental or external stress.

## 2. Results

### 2.1. Mutation of MCD1 and PDS5 Showed Major Markers of Apoptosis

**Figure 1:**
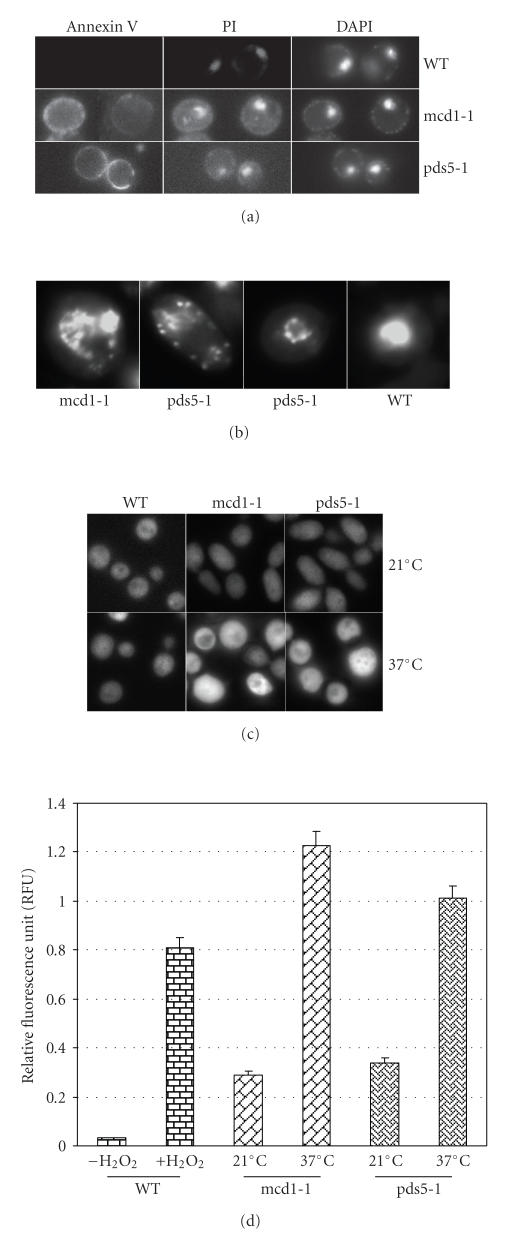
Apoptotic cell death in mcd1-1 and pds5-1 mutants. WT, mcd1-1, and pds5-1 mutants were shifted from permissive temperature (21°C) to nonpermissive temperature (37°C) for 12 hours, then stained with (a) Annexin V, PI, and DAPI; (b) DAPI for nucleus and (c) Dihydrorhodamine 123 for ROS. (d) Quantitative measurement of ROS production of H2O2-treated cells, mcd1-1 and pds5-1 mutants at permissive, and nonpermissive temperature.

**Figure 2:**
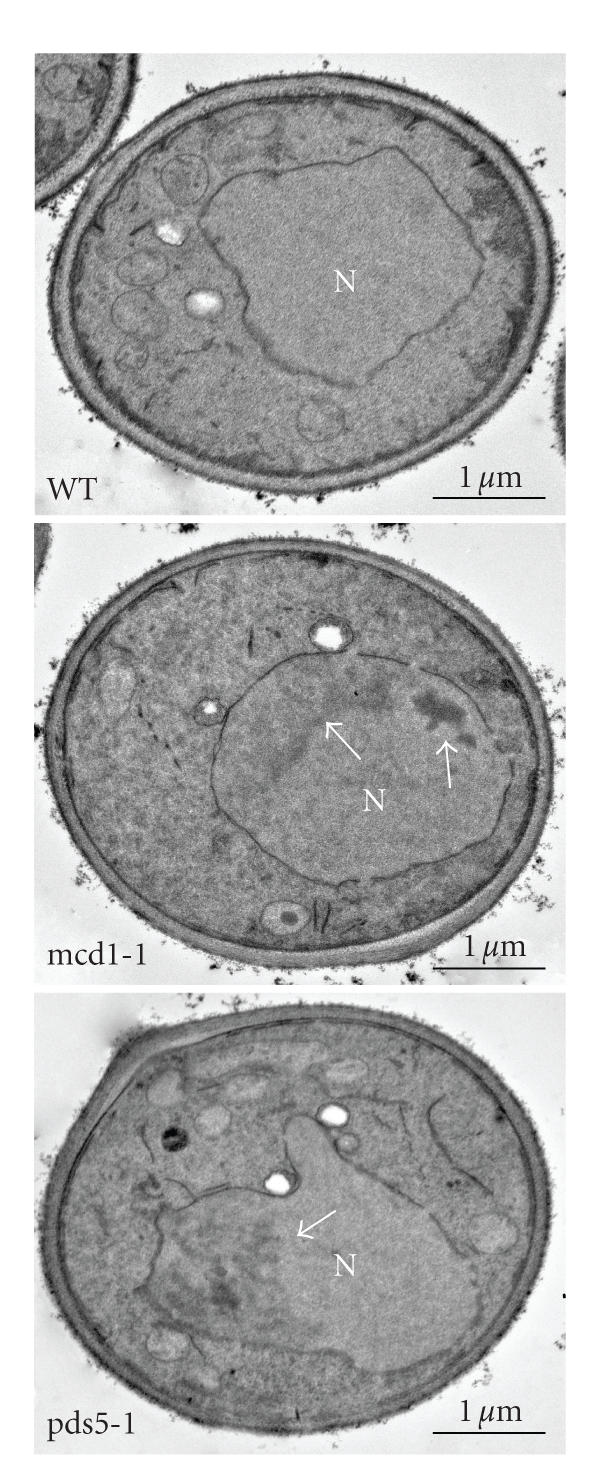
TEM images of mcd1-1 and pds5-1 mutants, growing at nonpermissive temperature for 2 hours, showing chromatin condensation (arrow in mcd1-1 and pds5-1) *N* = Nucleus.

Our previous report [7] showed that mutation of PDS5 caused apoptotic cell death in yeast during early meiosis. Here, we demonstrated that mutation of PDS5 and MCD1, two cohesin related genes, caused apoptotic cell death during mitosis. Figure 1 shows some of the “hallmarks” of apoptosis—the translocation of phosphatidylserine from the inner leaflet to the outer leaflet of the plasma membrane (Figure 1(a)), and nuclear fragmentation (Figure 1(b)). ROS accumulation was also observed in mcd1-1 and pds5-1 mutants, when grown at nonpermissive temperature (Figures 1(c) and 1(d)). Note that the ROS level was higher in mcd1-1 and pds5-1 mutants even at 21°C (Figure 1(d)), compared to the wild type.Chromatin condensation was also observed by transmission electron microscopy (Figure 2).

### 2.2. General Characteristics of Gene Expression

**Figure 3:**
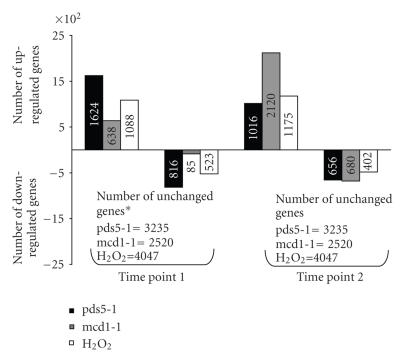
Number of up- and downregulated genes at time point 1 and point 2 in H_2_O_2_-treated cells, mcd1-1, and pds5-1 cells.*The number of unchanged genes includes genes with marginal increase and marginal decrease (see Materials and Methods for detail).

**Figure 4:**
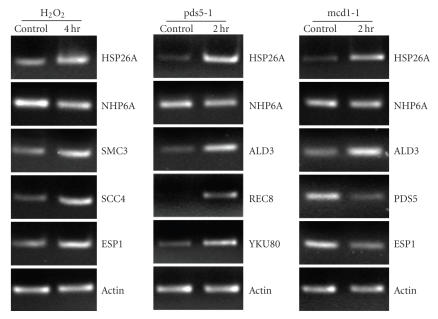
RT-PCR of 5 different genes from each group of time point 1.

**Figure 5:**
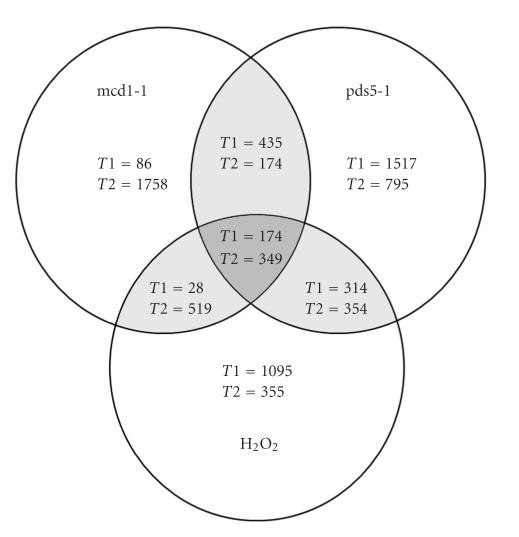
Schematic representation of differentially expressed genes in the three groups. *T*1: time point 1; *T*2: time point 2.

In attempt to understand the mechanism of the cohesininduced apoptosis in yeast,a global analysis of gene expression was performed in mcd1-1 and pds5-1 mutants undergoing apoptotic cell death. H_2_O_2_-treated cells were used as a positive control.The number of up- and downregulated genes were shown in Figure 3 (the original data is available at http://www.uwyo.edu/microscopy/cohesin.xls).The microarray data were further validated by reverse transcriptase-polymerase chain reaction (RT-PCR) (Figure 4). 5 different genes from each group were chosen for RT-PCR analysis.These genes represent genes that are up- and downregulated, and genes related to cohesin complex, which is of interest for further analysis. At both time points (see materials and methods for determination of the time points), more genes were upregulated than downregulated, suggesting a genetic program was being activated, rather than the general gene expression shutdown that might be expected in a rapid cell death. At time point 1, pds5-1 had more genes differentially regulated than the other two, while mcd1-1 had the least. At time point 2, however, the number of differentially regulated genes in mcd1-1 surpassed that of pds5-1 or H_2_O_2_-treated cells, especially the number of upregulated genes (Figures 3, 5). Furthermore, while the number of differentially regulated genes in pds5-1 and H_2_O_2_-treated cells decreased from time point 1 to time point 2, the number of genes in mcd1-1 increased from time point 1 to time point 2. These results suggest that cells may have a slower but more profound response at later time point, to the defect of Mcd1, than to the defect of Pds5, or H_2_O_2_-treated cells.

Only a small number of differentially expressed genes overlap among the three groups in both time points (Figure 5). The total number of overlapped genes were 174 in time point 1 (167 upregulated, and 7 downregulated) and 349 in time point 2 (323 upregulated and 26 downregulated). This suggests that these three groups may undergo apoptotic cell death through different pathways. To better understand the pathways, the gene expression profiles were analyzed using Netaffx analysis software from Affymetrix (http://www.affymetrix.com/). Using Affymetrix's gene ontology (GO) mining tool, it was found that in H_2_O_2_-treated cells, the most prominent group of genes (the group with the lowest *ρ*-value) that were differentially expressed was “response to stimulus” (Table 2). In mcd1-1 and pds5-1 mutants, however, the most prominent group is related either to physiological process (mcd1-1), or regulation of biological process (pds5-1). This may reflect the fact that the H_2_O_2_-treated cells mainly responded to the external stress, termed “environmental stress response” (ESR) [25], while in mcd1-1 and pds5-1 mutants, the gene expression responded mainly to biological or physiological changes due to the malfunction of MCD1 or PDS5.

### 2.3. Genes Involved in Response to Stimulus

When the 174 overlapped genes from the three groups in time point 1 were analyzed using the GO mining tool, it was found that the most prominent group of differentially expressed genes was related to “response to stimulus” (Table 2), suggesting that, like the H_2_O_2_-treated cells, the mcd1-1 and pds5-1 mutants, when grown at nonpermissive temperature, were also under stress. An evidence of the stress was the production of ROS (Figure 1). The changes of gene expression in mcd1-1 and pds5-1 mutants were referred to “internal stress response” (ISR), contrasting to the environmental, or external stress response (ESR). At time point 2, the most prominent change of gene expression in all types of cells, including H_2_O_2_-treated cells, was related to either physiological process (mcd1-1), or regulation of biological process (pds5-1 and H_2_O_2_-treated), suggesting that the ESR could eventually become ISR, if the stress continues.

Further analysis of the overlapped genes of the three groups revealed that at time point 1, 29 out of 174 of the overlapped genes were a response to stimulus, including response to DNA repair, oxidative stress, heat and nutrition (Table 3). Only 3 out of 29 were downregulated and 26 were upregulated. In time point 2, response to stimulus was one of the top three prominent groups that are differentially regulated (1st = regulation of biological process (Table 2), 2nd = development, 3rd = response to stimulus). Among the 58 differentially regulated genes response to stimulus, 31 (53%) were related to DNA damage (Table 4), suggesting that more DNA damages were caused by the extended time of internal or external stress. It has been reported that both Pds5 and Mcd1 are involved in DNA repair [26, 27]. The elevated number of differentially expressed genes response to DNA damage could be related in part, to the malfunction of mcd1-1 or pds5-1 in DNA damage repair.

**Figure 6:**
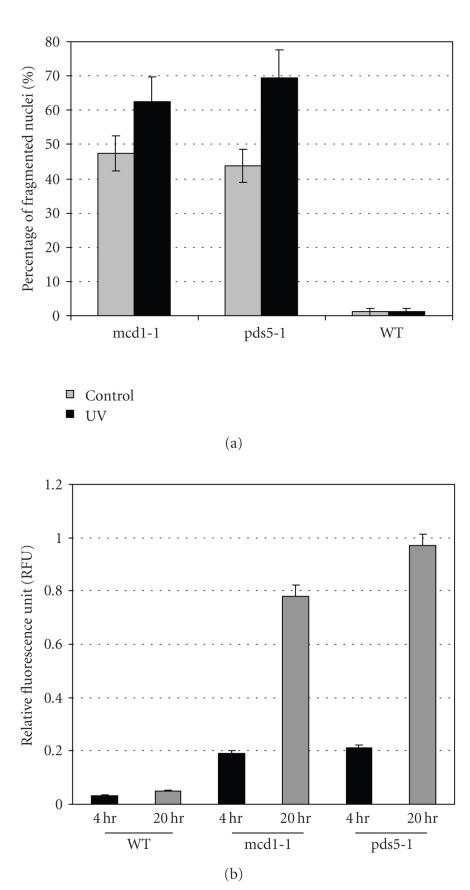
UV radiation caused increase of apoptotic cell death in mcd1-1 and pds5-1 mutants. (a) wild type, pds5-1 cells, and mcd1-1 cells were exposed to UV radiation (150 J/m^2^) then incubated for 20 hours at 37°C. Cells were stained with DAPI to identify the fragmented nuclei. Data are from 3 independent experiments; at least 100 cells were counted for each experiment. (b) ROS was produced in pds5-1 and mcd1-1 mutants. Wild type and the mcd1-1 and pds5-1mutants were exposed to UV radiation (150 J/m^2^) then incubated for 2 and 18 hours at 37°C. Dihydrorhodamine 123 (Priault et al. pages 684–91) was added into cell culture and incubated for another 2 hours, ROS production was measured using a microplate reader.

Previous reports indicate that both Pds5 [27, 28] and Mcd1 [26] have a role in DNA repair. To explore if the defect in DNA repair is involved in mcd1-1- or pds5-1-induced apoptosis, wild type, pds5-1- and mcd1-1 cells were exposed to UV radiation (150 J/m^2^) using a UV cross-linker (Stratagene, Inc.), and then incubated for 20 hours at 37°C before being assayed with DAPI. DAPI staining showed that in pds5-1, broken nuclei increased at least 18%, while in mcd1-1, broken nuclei increased about 15%, compared to cells without UV radiation. At the same condition, UV radiation had almost no effect on wild type (Figure 6(a)). ROS levels were much higher in mcd1-1 and pds5-1 mutants after UV radiation (Figure 6(b)).These results suggest that (1) Mcd1 and Pds5 participate in DNA break repair, and (2) defect of mcd1-1 or pds5-1 in DNA break repair plays a role in apoptosis.

### 2.4. Genes Involved Mitochondria Functions

Mitochondria play an important role in yeast apoptosis [29, 30]. In our current study, microarray analysis revealed that many differentially expressed genes were localized in mitochondria. In time point 1, among the 174 differentially expressed genes in all three groups, 38 were found localized in mitochondria (Table 5). 26 of the 38 genes were localized only in mitochondria. Interestingly, 12 of the 38 have unknown functions. The genes that have known functions were involved in response to stimulus, constituents of mitochondrial membrane, regulation of ATP syntheses, and other enzymes. Of particular interest is the upregulation of CYC7, which encodes the isoform 2 of Cytochrome C. Cytochrome C has been reported to be released from mitochondria to cytoplasm after induction of apoptosis [31]. The increase of expression level of CYC7 may also contribute to the release and the abundance of Cytochrome C in cytoplasm after induction of apoptosis. The upregulation of CYC7 only appeared in time point 1, not time point 2, suggesting that the release of Cytochrome C is an early event during apoptosis. At time point 2, 40 (12 localized only mitochondria; 8 with unknown functions) of the commonly up- or downregulated genes were found localized in mitochondria. Although the number of genes was similar to that of time point 1, the majority of genes were different and only 7 appeared in both time points (bold letters in Tables 5 and 6). Of the 7 genes, 3 (ELG1, NTG1, and NTH2) were response to stimulus/DNA repair; 3 (GCV1, GLT1, and AEP1) were related to amino acid/protein metabolism; and one (YDR332W) with unknown functions. In general, the mitochondrial genes were mainly involved in response to stimulus/DNA damage repair, metabolism of DNA, proteins, and amino acids. BIR1, the gene encodes an antiapoptotic protein, was found overexpressed in all three cases (Table 6). When checked at time point 1, BIR1 was also overexpressed in all three cases, but the fold change in mcd1-1 was less than 2, and therefore was filtered out. Because BIR1 is also involved in coordinating cell cycle events for proper chromosome segregation, it is unclear that the overexpression of BIR1 is due to its role of cell cycle control, or apoptosis.

### 2.5. Genes Involved in Cell Cycle Regulation

**Figure 7:**
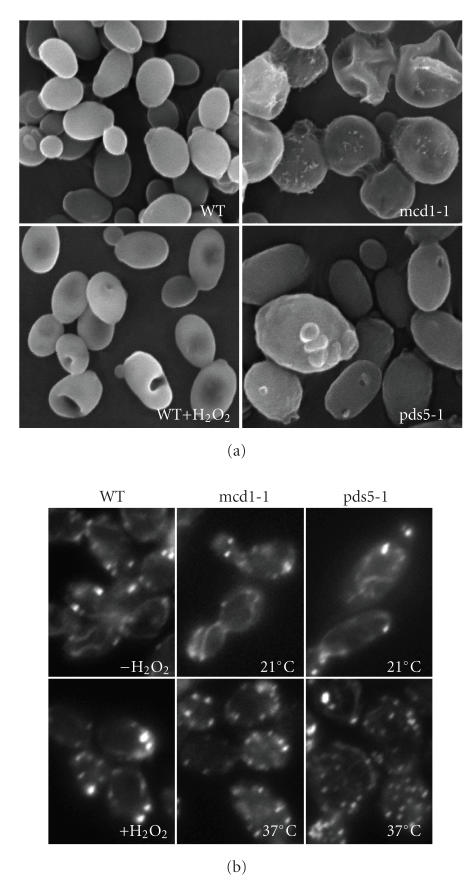
(a) Scanning electron microscopy images of wild type, H_2_O_2_-treated (4 hours) cells, mcd1-1 and pds5-1 cells at nonpermissive temperature for 2 hours. (b) rhodamine-phalloidin staining of actin of wild type (with or without H_2_O_2_ for 4 hours), mcd1-1 and pds51-1 mutants grown at permissive or nonpermissive temperature for 2 hours.

Deregulation of cell cycle has been reported in yeast cells undergoing apoptotic cell death [32, 33]. The apoptotic aging cells, for example, were described as to attempt the second budding cycle without the completion of the first one. Irregular expression of cell cycle related genes was also reported in old cells and orc2-1 cells that underwent apoptosis [33]. In our current study, we found that 62 genes that were differentially expressed in all three groups at time point 2 were related to cell cycle regulation (Table 7), while only 12 in time point 1 were related to cell cycle, suggesting that this deregulation occurs at the late stage of apoptosis. Cell cycle interruption was also observed at time point 1. In mcd-1-1 mutant, for example, SEM images showed the daughter cell was unable to bud off the mother cell even when the size of the daughter cell was similar to the size of the mother cell (Figure 7(a)). The 62 all upregulated genes (Table 7) were involved in both mitotic and meiotic cell cycles. 6 genes were involved in mitotic spindle checkpoint (MAD2, MAD3, DMA2, SGO1, IBD2, and MPS1); 3 in DNA damage checkpoint (PIN4, MEC3, LCD1) and two with unknown functions. Of the two genes with unknown functions, YOR019W interacts with ribosomes, and SPR6 was involved in sporulation. Stress, especially external (environmental, nutritional) stress, is the major cause of meiosis or sporulation [34]. The fact that 23 out of the 62 genes (Table 7) are involved in meiotic cell cycle suggests that yeast cells under the external (H_2_O_2_), or internal (mc1-1 and pds5-1) stress may attempt to initiate meiotic division. REC8, for example, is a meiotic specific gene that is only expressed during early meiosis. Its expression, however, was detected under all three conditions (Table 7). On the mitotic side, many of the differentially expressed genes are involved in checkpoints of G1/S or G2/M transitions, such as SWI4, a DNA binding component of the SBF complex (Swi4p-Swi6p) that regulates late G1-specific transcription of targets, including cyclins, and genes required for DNA synthesis and repair. Another example is SFI1, a Centrin (Cdc31p)-binding protein, required for progression through G2/M transition.

### 2.6. Genes Involved in Cell Wall Organization

Scanning electron microscopy was used to investigate the cell morphology under the external and internal stresses. Consistent with the microarray data, the cell surface structure of the pds5-1- and the H_2_O_2_-treated cells was similar, in which cell wall sunk, forming a hole on the cell surface (Figure 7(a)).The mcd1-1 cells appeared to be more damaged than pds5-1- or H_2_O_2_-treated cells. More cells collapsed, and cell surface was not as smooth as the other two, suggesting a potential cell composition change. The mcd1-1 cells were generally larger than the wild type or the other two. Further examination of the gene expression related to cell wall revealed that only 7 genes in time point 1 and 5 genes in time point 2 were differentially expressed in all three cases (Table 8), suggesting that the cytoskeleton, rather than the cell wall composition or organization, may contribute more to the morphological changes observed by SEM. To test the possibility of the involvement of cytoskeleton in cell-morphology change, rhodamine-conjugated phalloidin was used to stain actin. As shown in Figure 7(b), for the wild type and mutants grown at permissive temperature, actin appeared to be in both polymerized (filaments) and nonpolymerized (monomers) forms. The filamentous actin was usually oriented parallel to the longitudinal axis of the cell, as well as along the cell wall. In H_2_O_2_-treated cells, and mutants grown at nonpermissive temperature, actin was present mostly as monomers, indicating the damage of the cytoskeleton.

### 2.7. Apoptotic Cell Death at Nonpermissive Temperature

**Figure 8:**
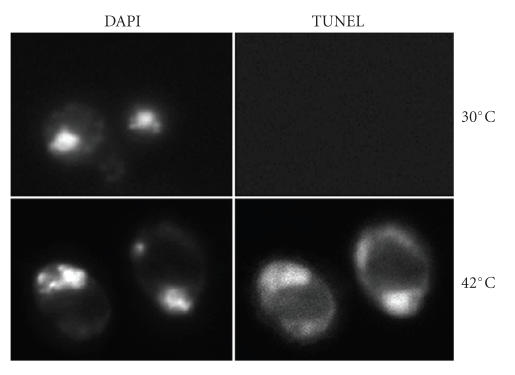
TUNEL staining of wild-type cells at permissive (30°C) and nonpermissive temperature (42°C), showing DNA breaks at nonpermissive temperature.

Temperature shift in several temperature-sensitive mutants, includes CDC48 [5], ORC2 [6], and PDS5 [7], showed apoptotic cell death. We speculate that all cells undergo apoptotic cell death at the nonpermissive temperature. To test this speculation, wild type cells were incubated overnight at 42°C, a nonpermissive temperature for wild type cells. Apoptotic cell death was revealed in majority of wild-type cells. More than 90% of wild-type cells showed positive Annexin V staining, nuclei fragmentation, and chromatin condensation. About 50% of cells showed positive TUNEL staining, while none showed positive TUNEL staining at permissive temperature (Figure 8). ROS level was much higher than cells grown at 30°C. Wild type showed no significant difference from the mcd1-1 or pds5-1 mutants when both incubated at 42°C (data not shown). This result suggests that high temperature stress causes apoptosis in yeast and can be used as an apoptosis inducer for studying the mechanism of apoptotic cell death in yeast.

## 3. Discussion

Sister chromatid cohesin plays a crucial role in accurate chromosomal segregation in the eukaryotic cell cycle. Recent studies indicate that cohesin proteins are also involved in apoptosis. Two studies showed that the Rad21 of mammalian cells was cleaved during apoptosis induced by diverse stimuli. The C terminal of Rad21 cleavage product was translocated to cytoplasm and caused amplification of the cell-death signal [21, 22]. In human breast cancer cells, expression level of RAD21 is higher than normal cells. Inhibition of RAD21 lows the survival rate of breast cancer cells and induces apoptotic cell death [24]. In S. cerevisiae, mutation of PDS5 causes apoptotic-like cell death in early meiosis [7]. When treated with H_2_O_2_, which causes apoptosis in yeast [3], the Mcd1 level was decreased (Yang & Zhang, unpublished data), suggesting a role of MCD1 in peroxide-induced apoptosis. Our current study further suggests that certain cohesin proteins, such as Mcd1 or Pds5, are required for cell proliferation. Malfunction of these cohesin proteins causes apoptotic cell death in yeast, as well as in mammalian cells.

Yeast cells show an apoptotic phenotype under a variety of environmental stresses, such as hydrogen peroxide [3, 4]. Temperature shift in several temperature-sensitive mutants includes CDC48 [5], ORC2 [6], and PDS5 [7], and also shows apoptotic cell death. Our current study suggests that the cell death caused by nonpermissive temperature is due to the internal stress response (ISR), compared to external stress response (ESR). A common feature shared by the ISR and ESR is response to stimulus, including response to DNA damage, oxidative stress, and heat. Our data also suggest that ESR may become ISR that ultimately causes the cell death. Since all the genes in the temperature sensitive mutants are essential genes, it would be interesting to see if cell death caused by the malfunction of all essential genes is apoptotic and due to ISR. Another commonality of the yeast cell death is the apoptotic nature of cell death at nonpermissive temperature, because all yeast strains, including the wild type die at their nonpermissive temperature, that is, above 37°C. Our result shows that even the wild-type cells undergo apoptotic cell death at their nonpermissive temperature (Figure 8).

Several studies have shown that DNA damage stimulates apoptosis in budding yeast. Mutation in CDC13, for example, triggers apoptotic cell death due to the accumulation of DNA damage [35]. UV radiation also induces some features of apoptosis [36]. These findings suggest that yeast may share some of the apoptotic pathways induced by DNA damage in mammals, especially those p53-independent pathways. Sister chromatid cohesin also appears to be involved in DNA repair. In fact, the cohesin subunit Rad21 was first identified in Schizosaccharomyces pombe by its sensitivity to UV or ionizing radiation, suggesting a role in DNA repair [37]. A recent study showed that downregulation of RAD21 impaired DNA double strand break repair and cells were more sensitive to etoposide and bleomycin, which induce double strand breaks [24]. In fission yeast, mutants of PDS5 lost the proliferative ability after arrest in *G*_2_ and were hypersensitive to DNA damage [27]. Our current study further confirms that DNA damage plays an important role in yeast cell death due to the fact that (1) a large amount of genes related to DNA damage repair are differentially expressed during apoptosis by either H_2_O_2_ treatment or by mutation of the cohesin protein Mcd1 or Pds5; and (2) UV radiation caused significant increase of cell death in mcd1-1 and pds5-1 mutants. We speculate that this defect in DNA repair may trigger the signaling pathway that ultimately leads to the cell death.

## 4. Materials and methods

### 4.1. Yeast Strains and Culture Conditions

The wild-type strain used in this study is *MATa ADE2 ade5 can*1^*R*^ *CYH*2^*s*^ *his7 2 leu1-d lys2-1 met13-d trp1-63 tyr1-1 ura3-13*. Plasmids of mcd1-1 and pds5-1 were provided by Dr. V Guacci and transformed into the wild-type strain. The construction and characterization of both mcd1-1 and pds5-1 were described previously [15, 38]. Both mcd1-1 and pds5-1 are temperature sensitive mutants, growing normally at 21°C, but unable to grow at 37°C. Cells were grown in YPDA medium (1% Yeast Extract, 2% Peptone, 2% Dextrose, 0.001% Adenine) on a mechanical shaker at specified temperature.

### 4.2. Annexin V Staining

Exposed phosphatidylserine was detected by Alexa Fluor-488 conjugated annexin V (Invitrogen, Inc.). 1 × 10^5^ yeast cells were collected and washed twice with sorbitol buffer (0.8 M sorbitol, 2% potassium acetate, pH 7.0), resuspended in sorbitol buffer containing 10 mM dithiothreitol for 10 minutes, then digested with 0.4 mg mL^−1^ Zymolyase 100 T (ICN Biomedicals, Inc.) for 30 minutes. Cells were then harvested and resuspended in 50 *μ*L binding buffer (10 mM HEPES, 140 mM NaCl, 2.5 mM CaCl_2_, 1.2 M Sorbitol, pH 7.4). 4 *μ*L of annexin V conjugate, 2 *μ*l of 1 *μ*g mL^−1^ propidium iodide (PI) were added to the cell suspension and incubated for 20 minutes at room temperature in dark. Cells were rinsed with binding buffer, mounted under a coverslip with antifading medium (0.1 M propyl gallate, 50% glycerol in PBS) containing 0.5 *μ*g mL^−1^ 4’, 6-diamidino-2-phenylindole (DAPI). A Nikon-TE300-inverted microscope, equipped with a Cascade 650-cooled CCD monochrome camera (Roper Scientific) was used for image acquisition.

### 4.3. Terminal Deoxynucleotidyl Transferase-Mediated DUTP Nick End Labeling (TUNEL) Assay

DNA breaks were detected by the ApoAlert DNA Fragmentation Assay Kit (Clontech Laboratories, Inc.) following the manufacturer's user manual. Briefly, yeast cells were digested with 0.1 mg/mL Zymolyase 100 T for 5 minutes, then spread on polylysine-coated slides. The slides were fixed with 4% paraformaldehyde for 30 minutes, and then rinsed with PBS. Cells were permeabilized by 0.2% Triton X-100 in PBS for 5 minutes on ice. DNA breaks were detected by TUNEL reaction using fluorescein conjugated dUTP. Positive control is conducted by incubation of cells with DNase I and negative control by omitting the terminal deoxynucleotidyl transferase.

### 4.4. Transmission Electron Microscopy

TEM sample preparation was conducted as described previous [34]. Briefly, cells were fixed with 2.5% (v/v) glutaraldehyde in phosphate buffered saline (PBS) (pH = 7.2) for 40 minutes at room temperature. Cells were further fixed with 2% potassium permanganate in water for 1 hour at room temperature, then stained with 2% uranyl acetate for 90 minutes. Fixed cells were dehydrated with 30%, 50%, 75%, 85%, 95%, and 100% ethanol, then embedded in Spurr resin (Electron Microscopy Sciences, Pa). 60 nm ultrathin sections were cut with a diamond knife, stained with lead citrate for 30 minutes, and examined using a Hitachi H-7000 electron microscope, equipped with a high-resolution (4 K × 4 K)-cooled CCD digital camera (Gatan, Inc., Pa, USA).

### 4.5. Detection of Reactive Oxygen Species (ROS)

ROS was detected with dihydrorhodamine 123 (Sigma Chemical Co., Mo, USA). Dihydrorhodamine 123 (50 *μ*M, final concentration) was added into cell culture and incubated for 2 hours. Cells were then mounted on microscope slides and viewed with fluorescence microscope using a rhodamine optical filter. A GEMINI XS fluorescence microplate reader (Molecular Devices, CA) was used for the quantitative measurement of ROS. H_2_O_2_ treated cells, mcd1-1, pds5-1 mutants were stained with Dihydrorhodamine 123 and placed in a 96 well plate. Fluorescence reading was perfumed with the wavelength of 570 nm for excitation and 615 nm for emission.

### 4.6. Microarray Analysis

For pds5-1 cells and mcd1-1 mutants, cells were inoculated into fresh YPDA medium and cultured at 21°C overnight to a density of 1.0 × 10^7^ cells/ml, then shifted to 37°C for 2 or 12 hours and harvested for RNA isolation. The 2- and 12-hour time points were chosen because these time points represented cells with an apoptosis rate of 1–5% and 50–60%, respectively. These two time points were referred as time point 1 (2 hours) and time point 2 (12 hours). For control, wild-type cells were treated as the same as above and RNA was isolated at the two same time points.

H_2_O_2_-treated wild-type cells were used as a positive control. Wild-type cells were inoculated into fresh medium and incubated at 37°C until the cultures reached a density of 5 × 10^6^ cells/mL. 5 mM H_2_O_2_ was added to the culture and the cells were continually cultured at 37°C for 4 or 10 hours before harvested for total RNA isolation. The 4- and 10-hour treatment of H_2_O_2_ represents an apoptosis rate of 3–5% and 50–60%, respectively, and as referred to time point 1 and time point 2, corresponding to mcd1-1 and pds5-1. The control sample was treated as same as above, except with the omission of H_2_O_2_.

Total RNA was extracted using the enzymatic lysis protocol of Qiagen RNeasy minikits (Qiagen; Valencia, Calif) with the following modification. Buffer Y1 was replaced with sorbitol buffer (0.8 M sorbitol, 2% potassium acetate, pH 7.0). 0.5 mg/mL (final concentration) of Zymolyase-100 T (MP Biomedicals, Aurora, Ohio) was used for cell-wall removal and the enzyme digestion time was 5 minutes. RNA labeling and hybridization procedures were performed according to protocols from the Microarray Core at the University of Colorado Health Science Center.

At each time point, total RNA was extracted from 5–10 × 10^7^ cells using 2 Qiagen columns. The quality of the purified total RNA was evaluated by Agilent bioanalyzer and NanoDrop Spectrophotometer. 2.5 *μ*g of high-quality total RNA was used as template for cDNA synthesis by reverse transcription. Affymetrix one-cycle cDNA Synthesis Kit, cDNA Sample Cleanup Module, and IVT Labeling Kit were used to produce biotinylated cRNA target. The Agilent bioanalyzer and NanoDrop Spectrophotometer were used to determine the cRNA yield, purity, and size distribution. Approximately, 20 *μ*g of cRNA were used for fragmentation with the buffer from Affymetrix Hybridization Control Kit. The fragmented biotin-labeled cRNA was quantitated, and hybridized to the Affymetrix GeneChip Yeast Genome 2.0 Array, which includes approximately 5 744 probe sets for 5 841 of the 5 845 genes present in S. cerevisiae.

Microarray data was first analyzed using Affymetrix's GeneChip Operating Software. The expression level of the cohesin mutants and H_2_O_2_-treated cells at different time points was compared with that of the corresponding control cells, using the software's default parameters (Alpha1 = 0.05, Alpha2 = 0.065, Tau = 0.015, Gamma1H = 0.002, Gamma1L = 0.002, Gamma2H = 0.002667, Gamma2L = 0.002667, Perturbation = 1.1). A call to each gene indicating the change in transcript level between the baseline (control) and the experiment array was assigned with one of the following values: Increase (I), Decrease (D), No Change (NC), Marginal Increase (MI), or Marginal Decrease (MD). Genes with a value of NC, MI, or MD were all regarded as no change of transcription level, and only the genes with a value I or D were selected. Fold change of the differentially expressed genes was calculated based on the signal-log ratio. Only the genes with a fold change equal to or greater than 2 were selected to ensure the gene expression was truly differential. Microarray data were derived from two independent experiments. Values were average of the two-independent experiments

### 4.7. Scanning Electron Microscopy (SEM)

Cells at time point 1 were fixed with 2.5% glutaraldehyde in phosphate buffer (pH 7.3), then dehydrated with ethanol (30, 50, 75, 85, 95, 100%, 5 minutes each). Cells were dried with a critical point dryer; sputter coated with gold, and examined using a JEOL JSM5800LV SEM, operated at 20 Kv.

### 4.8. Rhodamine-Phalloidin Staining of Actin

Cells at time point 1 were fixed in PBS buffer with 4% formaldehyde for 1 hour. After washing with PBS buffer three times, fixed cells were resuspended in 1 mL PBS containing 1% Triton X-100 and incubated at room temperature for 3 minutes for cell permeabilization. Permeabilized cells were washed with PBS buffer, resuspended in 20 *μ*l rhodamine-phalloidin (Invitrogen) (final concentration = 160 nM) for 1 hour. Cells were mounted on a glass slide and viewed with a rhodamine filter.

## 5. Conclusion

Our data suggests that the cell death caused by mutation of MCD1 or PDS5 is due to the internal stress response, contrasting to the environmental or external stress response induced by external stimuli, such as hydrogen peroxide. A common feature shared by the internal stress response and external stress response is the response to stimulus, including response to DNA damage, mitochondria functions, and oxidative stress, which play an important role in yeast apoptotic cell death.

## Figures and Tables

**Table 1:** Fold changes of each gene expression, corresponding to the RT-PCR in Figure 4.

H_2_O_2_		pds5-1		mcd1-1	
GENE	Fold change	GENE	Fold change	GENE	Fold change
HSP26A	+10.6	HSP26A	+30	HSP26A	+9.8
NHP6A	−4	NHP6A	−2.3	NHP6A	−2
SMC3	+8	ALD3	+52	ALD3	+12
SCC4	+9.8	REC8	+19.7	PDS5	−1.7
ESP1	+6.1	YKU80	+7.5	ESP1	−1.3
Actin	Control	Actin	Control	Actin	Control

**Table 2:** The most prominent gene group (the group with the lowest *ρ*-value) that is differentially expressed in response to the H_2_O_2_ treatment, or MCD1 and PDS5 mutations.

	Time point 1	Time point 2
mcd1-1	Physiological process (GO ID = 7582)	Physiological process (GO ID = 50789)
pds5-1	Regulation of biological process (GO ID = 50789)	Regulation of biological process (GO ID = 50789)
H_2_O_2_	Response to stimulus (GO ID = 50896)	Regulation of biological process (GO ID = 50789)
All three	Response to stimulus (GO ID = 50896)	Regulation of biological process (GO ID = 50789)

**Table 3:** List of differentially regulated genes response to stimulus in time point 1.

Response to	Gene	Fold change			Description
		+H_2_O_2_	mcd1-1	pds5-1	
DNA damage	RMI1	10.56	2.83	9.85	Involved in response to DNA damage
	RFX1	8.57	2.14	6.50	Involved in DNA damage checkpoint pathway
	EPL1	7.46	2.14	6.96	Component of NuA4, a histone H4/H2A acetyltransferase complex
	NTG1	6.96	2.30	7.46	DNA N-glycosylase and apurinic/apyrimidinic (AP) lyase
	YKU80	6.96	2.14	7.46	Subunit of the telomeric Ku complex (Yku70p-Yku80p)
	*NHP6A*	−4.00	−2.00	−2.30	High-mobility group nonhistone chromatin protein

oxidative stress	SRX1	21.11	3.73	9.19	Sulfiredoxin
	HSP12	11.31	2.46	8.57	Plasma membrane localized protein that protects membranes from desiccation
	CTT1	10.56	4.92	17.15	Cytosolic catalase T
	GRE3	8.57	2.46	13.00	Aldose reductase

Heat	HSP26	10.56	9.85	29.86	Small heat shock protein with chaperone activity
	GRE3	8.57	2.46	13.00	Aldose reductase
	SSA3	8.00	2.46	12.13	ATPase involved in protein folding and the response to stress
	SSA4	8.00	2.14	11.31	Heat shock protein that is highly induced upon stress
	SSE2	6.50	2.00	8.00	Member of the heat shock protein 70 (HSP70) family
	NTH2	13.93	2.00	10.56	Putative neutral trehalase

nutrition & others	ALD3	19.70	12.13	51.98	Cytoplasmic aldehyde dehydrogenase
	PTR3	9.19	3.03	12.13	Component of the SPS plasma membrane amino acid sensor system
	DDR2	8.57	6.50	17.15	Multistress response protein
	FIG2	8.57	3.25	8.57	Cell-wall adhesin
	ADR1	8.57	3.03	9.85	Carbon source-responsive zinc-finger transcription factor
	YGP1	8.00	3.03	10.56	Cell-wall-related secretory glycoprotein
	HAL1	7.46	2.83	6.50	Cytoplasmic protein involved in halotolerance
	TSL1	6.96	4.29	13.93	Large subunit of trehalose 6-phosphate synthase/phosphatase complex
	MET4	6.50	2.46	9.19	Lecine-zipper transcriptional activator
	MPT5	6.50	2.00	9.19	Member of the Puf family of RNA-binding proteins
	*PHO5*	−2.46	−2.14	−2.00	Repressible acid phosphatase
	*TIR1*	−2.83	−2.64	−2.00	Cell wall mannoprotein of the Srp1p/Tip1p family of serine-alanine-rich proteins

Unknown	YIL169C	10.56	3.73	27.86	Putative protein of unknown function
	YOR019W	9.19	2.46	10.56	Hypothetical protein

**Table 4:** List of differentially expressed genes in time point 2 response to DNA damage (bold = genes differently expressed in both time points).

Gene	Fold change			Description
	H_2_O_2_	mcd1-1	pds5-1	
PSO2	14.93	6.96	9.19	Required for a postincision step in repair of DNA breaks
CST9	13.93	4.00	14.93	SUMO E3 ligase
EAF7	13.00	2.46	9.85	Subunit of the NuA4 histone acetyltransferase complex
MIG3	12.13	4.29	14.93	Probable transcriptional repressor
**EPL1**	**12.13**	**3.73**	**8.57**	Component of NuA4
**NHP6A**	**12.13**	**3.73**	**8.57**	High-mobility group non-histone chromatin protein
SCC4	12.13	2.30	9.19	Subunit of cohesin loading factor (Scc2p-Scc4p)
SLX1	11.31	2.83	10.56	Subunit of 5'-flap endonuclease complex
VID21	10.56	4.59	13.00	Component of the NuA4 histone acetyltransferase complex
**YKU80**	**10.56**	**3.25**	**9.85**	Subunit of the telomeric Ku complex (Yku70p-Yku80p)
RAD59	9.19	6.50	9.19	Involved in repair of DNA double-strand breaks
HPR5	9.19	2.83	9.19	DNA helicase and DNA-dependent ATPase
SIR4	9.19	2.83	7.46	Silent information regulator that,
EAF6	9.19	2.46	7.46	Esa1p-associated factor, subunit of the NuA4 acetyltransferase complex
TFB3	9.19	2.14	9.19	Subunit of TFIIH and nucleotide excision repair factor 3 complexes
**NTG1**	**8.57**	**3.48**	**7.46**	DNA N-glycosylase and apurinic/apyrimidinic (AP) lyase
MGT1	8.57	2.64	9.19	DNA repair methyltransferase
SNF5	8.57	2.64	9.19	Subunit of the SWI/SNF chromatin remodeling complex
HMI1	8.57	2.46	6.96	Mitochondrial inner-membrane localized ATP-dependent DNA helicase
RTT107	8.57	2.00	7.46	Interacts with Mms22p and is implicated in Mms22-dependent DNA repair
EXO1	8.00	2.64	8.00	5'-3' exonuclease and flap-endonuclease
ELC1	8.00	2.14	11.31	Elongin C
HEX3	8.00	2.14	6.96	Protein containing a RING finger domain that interacts with Slx8p
DOA1	8.00	2.00	8.57	WD repeat protein
MMS4	8.00	2.00	8.00	Subunit of the structure-specific Mms4p-Mus81p endonuclease
RAD16	7.46	5.28	8.00	Recognizes and binds damaged DNA in an ATP-dependent manner
MEC3	7.46	2.83	6.96	DNA damage and meiotic pachytene checkpoint protein
PIN4	7.46	2.46	9.19	Involved in G2/M phase progression and response to DNA damage
CAC2	7.46	2.00	8.57	Component of the chromatin assembly complex
LCD1	6.96	3.48	7.46	Essential protein required for the DNA integrity checkpoint pathways
PAN2	6.96	2.30	12.13	Subunit of the Pan2p-Pan3p poly(A)-ribonuclease complex

**Table 5:** List of differentially regulated genes that are related to mitochondria functions in time point 1 (Bold = genes appeared in both time points;* = only those localized in mitochondria).

	Gene name	Fold change			Description
		H_2_O_2_	mcd1	pds5-1	
Localized only in mitochondria	CYC7	13.93	2.64	9.85	Cytochrome c isoform 2
	OM14	11.31	3.25	14.93	Integral mitochondrial outer membrane protein
	ALD4	9.19	2.64	19.70	Mitochondrial aldehyde dehydrogenase
	LSC2	8.57	2.30	9.85	Beta subunit of succinyl-CoA ligase
	CEM1	8.57	2.30	11.31	Mitochondrial beta-keto-acyl synthase
	STF1	8.57	2.46	9.85	Involved in regulation of the mitochondrial F1F0-ATP synthase
	RSM19	8.00	2.14	8.00	Mitochondrial ribosomal protein of the small subunit
	OM45	8.00	3.03	16.00	Major constituent of the mitochondrial outer membrane
	CYB2	8.00	4.00	12.13	Cytochrome b2 (L-lactate cytochrome-c oxidoreductase)
	YME2	7.46	2.00	7.46	Integral inner mitochondrial membrane protein
	MRPL19	7.46	2.00	8.57	Mitochondrial ribosomal protein of the large subunit
	SLM5	7.46	2.14	9.19	Mitochondrial asparaginyl-tRNA synthetase
	**AEP1**	6.96	2.00	7.46	Required for expression of the mitochondrial OLI1 gene
	FMP46	6.96	2.00	10.56	Putative redox protein containing a thioredoxin fold
	MBR1	6.96	3.73	9.85	Involved in mitochondrial functions and stress response
	ODC1	6.06	2.00	9.19	Mitochondrial inner membrane transporter
	**GLT1**	−3.48	−2.00	−2.46	NAD(+)-dependent glutamate synthase

Localized in mitochondria and nucleus/cytoplasm	**NTH2**	13.93	2.00	10.56	Putative neutral trehalase
	**GCV1**	11.31	2.83	12.13	T subunit of the mitochondrial glycine decarboxylase complex
	GOR1	9.85	2.00	10.56	Glyoxylate reductase
	DIA4	9.19	2.30	8.57	Probable mitochondrial seryl-tRNA synthetase
	RSF1	8.00	2.30	8.57	Protein required for respiratory growth
	YHL009W-B	8.00	2.83	8.57	Retrotransposon TYA Gag and TYB Pol genes
	**NTG1**	6.96	2.30	7.46	DNA N-glycosylase and apurinic/apyrimidinic (AP) lyase
	**ELG1**	6.96	2.30	10.56	Required for S phase progression and telomere homeostasis
	LRG1	−2.46	−2.30	−3.25	Putative GTPase-activating protein (GAP)

Of unknown functions	TMA10	19.70	7.46	13.93	Protein of unknown function that associates with ribosomes
	*YPL222W	12.13	2.30	9.85	The authentic, nontagged protein was localized to the mitochondria.
	*YDR379C-A	10.56	2.83	10.56	Hypothetical protein identified by homology.
	UIP4	8.57	2.64	19.70	Protein of unknown function that interacts with Ulp1p
	*YDR070C	8.57	9.85	22.63	The authentic, nontagged protein was localized to the mitochondria
	*YOR205C	8.00	2.00	9.85	The authentic, nontagged protein was localized to the mitochondria
	*YHL021C	7.46	2.14	6.96	The authentic, nontagged protein was localized to the mitochondria
	*YDR332W	7.46	2.64	11.31	Hypothetical protein
	YML128C	7.46	6.06	22.63	Protein of unknown function
	*YJL062W-A	6.96	2.14	8.57	Putative protein of unknown function
	*YNR040W	6.96	2.30	8.57	Hypothetical protein
	*YNL195C	6.96	3.03	27.86	Hypothetical protein

**Table 6:** List of differentially expressed genes that are related to mitochondria functions in time point 2, (bold = genes appeared in both time points;* = only those localized in mitochondria).

	Gene name	Fold change			Description
		H_2_O_2_	mcd1-1	pds5-1	
Localized only in mitochondria	CRC1	27.86	32.00	9.85	Mitochondrial inner membrane carnitine transporter
	PUT1	16.00	32.00	8.57	Proline oxidase
	HXT14	13.00	9.19	9.85	Hexose transport
	UBP16	11.31	2.46	8.57	Ubiquitin-dependent protein catabolism
	AAC1	11.31	8.00	8.57	Mitochondrial inner membrane ADP/ATP translocator
	PGS1	10.56	2.83	7.46	Phosphatidylglycerolphosphate synthase
	YHL009W-A	10.56	3.48	9.19	DNA-mediated transposition
	GCV2	9.19	2.64	12.13	P subunit of the mitochondrial glycine decarboxylase complex
	**AEP1**	9.19	3.48	11.31	Protein biosynthesis
	HTD2	9.19	3.73	8.00	Mitochondrial 3-hydroxyacyl-thioester dehydratase
	HMI1	8.57	2.46	6.96	Mitochondrial ATP-dependent DNA helicase
	ARG2	7.46	2.64	9.85	Acetylglutamate synthase
	**GLT1**	−2.64	−3.48	−2.64	NAD(+)-dependent glutamate synthase
	ILV5	−3.03	−2.14	−3.48	Acetohydroxyacid reductoisomerase
	POR2	−3.03	−2.00	−2.00	Ion transport

Localized in mitochondria and nucleus/cytoplasm	JJJ1	16.00	2.14	21.11	endocytosis
	HSF1	12.13	3.73	9.19	Trimeric heat shock transcription factor
	SPC105	9.85	3.03	7.46	Protein required for accurate chromosome segregation
	**GCV1**	9.85	8.00	8.00	T subunit of the mitochondrial glycine decarboxylase complex
	**NTH2**	9.19	5.28	9.19	Putative neutral trehalase
	VPS15	8.57	2.00	7.46	Myristoylated serine/threonine protein kinase
	VPS54	8.57	2.00	8.00	Component of the Golgi-associated retrograde protein complex
	PTC5	8.57	2.00	8.57	Mitochondrially localized type 2C protein phosphatase
	LAS1	8.57	2.00	12.13	Essential nuclear protein possibly involved in morphogenesis
	BIR1	8.57	2.46	8.57	Cell cycle, anti-apoptotic
	**ELG1**	8.57	2.46	12.13	Required for S phase progression and telomere homeostasis
	**NTG1**	8.57	3.48	7.46	DNA N-glycosylase and apurinic/apyrimidinic (AP) lyase
	AFT2	8.57	3.73	8.57	Iron-regulated transcriptional activator
	RIS1	8.00	2.46	7.46	Member of the SWI/SNF family of DNA-dependent ATPases
	GYP1	8.00	3.03	8.57	Vesicle-mediated transport
	SUR7	−2.83	−2.14	−3.48	Putative integral membrane protein
	MRH1	−2.83	−2.00	−2.83	Protein that localizes primarily to the plasma membrane

Of unknown functions	*YNL130C-A	18.38	8.57	12.13	Protein of unknown function
	*YBR047W	13.93	4.29	8.57	Protein of unknown function
	*YLR346C	11.31	11.31	14.93	Protein of unknown function
	*YLR253W	9.85	2.46	8.00	Protein of unknown function
	TOF2	9.85	3.03	13.93	Protein of unknown function
	*YDR332W	9.85	4.92	7.46	Hypothetical protein
	*YGL226W	8.57	2.30	8.00	Protein of unknown function
	*YOR305W	8.00	2.00	9.19	Protein of unknown function

**Table 7:** List of differentially regulated genes that are related to cell cycle in time point 2 (note that all genes are upregulated).

Gene name	Fold change			Description
	H_2_O_2_	mcd1-1	pds5-1	
CSM4	25.99	13.00	13.93	Meiotic chromosome segregation
REC8	10.10	14.90	8.60	Meiotic sister chromatid cohesion
SPR3	18.38	18.38	13.00	Cellular morphogenesis during conjugation with cellular fusion
REC114	17.15	4.92	22.63	Meiotic recombination
MER1	17.15	2.83	18.38	Regulation of nuclear mRNA splicing
CST9	13.93	4.00	14.93	Synaptonemal complex formation
SWI4	13.93	3.73	9.19	G1/S transition of mitotic cell cycle
SPS4	13.00	4.59	7.46	DNA metabolism
OKP1	13.00	2.14	8.57	Chromosome segregation
SFI1	12.13	3.73	11.31	G2/M transition of mitotic cell cycle
EPL1	12.13	3.73	8.57	Regulation of transcription from RNA polymerase II promoter
MPS3	12.13	2.83	10.56	Nuclear migration during conjugation with cellular fusion
SCC4	12.13	2.30	9.19	Double-strand break repair
MAD2	12.13	2.00	11.31	Mitotic spindle checkpoint
FKH2	11.31	3.48	13.00	G2/M-specific transcription in mitotic cell cycle
ISC10	11.31	2.64	14.93	Protein required for sporulation
MPC54	10.56	21.11	17.15	Spore wall assembly
DMA2	10.56	4.29	9.85	Establishment of mitotic spindle orientation
REC107	10.56	3.25	9.85	Meiotic recombination
IBD2	10.56	3.03	17.15	Mitotic spindle checkpoint
MPT5	10.56	2.64	12.13	Reentry into mitotic cell cycle after pheromone arrest
MAD3	10.56	2.64	9.85	Mitotic spindle checkpoint
CLN3	10.56	2.30	9.85	G1 cyclin involved in cell cycle progression
REC104	9.85	14.93	9.85	Meiotic recombination
KEL2	9.85	5.66	7.46	Regulation of exit from mitosis
KAR3	9.85	4.92	10.56	Mitotic sister chromatid cohesion
BCK2	9.85	3.25	9.19	Regulation of progression through cell cycle
RTT101	9.85	2.83	9.85	Negative regulation of DNA transposition
SLK19	9.85	2.46	7.46	Meiosis/mitotic spindle organization
LRS4	9.85	2.30	10.56	Protein involved in rDNA silencing
MND1	9.19	68.59	6.96	Meiotic recombination
HOP2	9.19	4.92	14.93	Meiotic recombination
KIP1	9.19	3.03	7.46	Mitotic spindle assembly and chromosome segregation
SLD2	9.19	2.46	9.19	DNA strand elongation
TFB3	9.19	2.14	9.19	Regulation of transcription
TAF2	9.19	2.14	9.19	G1-specific transcription in mitotic cell cycle
SPO11	8.57	7.46	8.57	DNA metabolism
PRP46	8.57	3.73	11.31	Nuclear mRNA splicing
UME6	8.57	3.25	9.85	G2/M transition of mitotic cell cycle
ELG1	8.57	2.46	12.13	Regulation of DNA transposition
CDC45	8.57	2.46	9.19	Prereplicative complex formation and maintenance
BIR1	8.57	2.46	8.57	Mitotic spindle elongation
CHL1	8.57	2.46	8.00	Mitotic sister chromatid cohesion
SGO1	8.57	2.30	8.57	Sister chromatid segregation
SLD3	8.57	2.14	9.19	DNA unwinding during replication
CEF1	8.00	3.73	10.56	Nuclear mRNA splicing
MPS1	8.00	3.25	8.57	Mitotic spindle assembly
SET3	8.00	3.25	7.46	Histone deacetylation
SPC72	8.00	2.64	8.00	Mitotic spindle elongation
CDC26	8.00	2.46	18.38	Protein ubiquitination
MCM22	8.00	2.30	9.85	Chromosome segregation
MMS4	8.00	2.00	8.00	Involvement in recombination and DNA repair
NIP100	7.46	4.92	9.85	Establishment of mitotic spindle orientation
DBF4	7.46	3.25	9.19	Regulation of DNA replication
MEC3	7.46	2.83	6.96	DNA damage checkpoint
PIN4	7.46	2.46	9.19	G2/M transition of mitotic cell cycle
GIC2	7.46	2.14	9.19	Regulation of exit from mitosis
IME1	6.96	17.15	16.00	Regulation of transcription
LCD1	6.96	3.48	7.46	DNA damage checkpoint
ULP1	6.96	2.46	7.46	Ubl (ubiquitin-like protein)-specific protease
YOR019W	13.93	8.57	11.31	Protein of unknown function
SPR6	10.56	2.30	9.19	Protein of unknown function

**Table 8:** List of differentially expressed genes in all three groups that are related to cell wall.

	Gene name	Fold change			Description
		H_2_O_2_	mcd1-1	pds5-1	
Time point 1	SSP2	10.56	3.03	10.56	Spore wall assembly
	SPI1	9.19	5.66	19.70	Cell-wall protein of unknown function
	FIG2	8.57	3.25	8.57	Cellular morphogenesis
	YGP1	8.00	3.03	10.56	Amino acid metabolism (response to stress/nutrient)
	GAS4	7.46	2.14	7.46	Carbohydrate metabolism
	PHO5	−2.46	−2.14	−2.00	Phosphate metabolism
	TIR1	−2.83	−2.64	−2.00	Cell-wall mannoprotein of the Srp1p/Tip1p family

Time point 2	SPR3	18.38	18.38	13.00	Cell-wall organization and biogenesis
	SSP2	16.00	9.19	9.85	Spore-wall assembly
	GAS4	11.31	4.59	8.57	Carbohydrate metabolism
	LRE1	6.96	2.14	7.46	Cell-wall organization and biogenesis
	SUR7	−2.83	−2.14	−3.48	Endocytosis
